# Mapping Interactions between mRNA Export Factors in Living Cells

**DOI:** 10.1371/journal.pone.0067676

**Published:** 2013-06-24

**Authors:** I-Fang Teng, Stuart A. Wilson

**Affiliations:** Department of Molecular Biology and Biotechnology, University of Sheffield, Firth Court, Western Bank, Sheffield, United Kingdom; University of Toronto, Canada

## Abstract

The TREX complex couples nuclear mRNA processing events with subsequent export to the cytoplasm. TREX also acts as a binding platform for the mRNA export receptor Nxf1. The sites of mRNA transcription and processing within the nucleus have been studied extensively. However, little is known about where TREX assembly takes place and where Nxf1 is recruited to TREX to form the export competent mRNP. Here we have used sensitized emission Förster resonance energy transfer (FRET) and fluorescence lifetime imaging (FLIM)-FRET, to produce a spatial map in living cells of the sites for the interaction of two TREX subunits, Alyref and Chtop, with Nxf1. Prominent assembly sites for export factors are found in the vicinity of nuclear speckles in regions known to be involved in transcription, splicing and exon junction complex formation highlighting the close coupling of mRNA export with mRNP biogenesis.

## Introduction

The TREX complex couples transcription and nuclear processing of mRNA with its subsequent export to the cytoplasm [1]. Once assembled on mRNA, TREX has the ability to release the RNA binding domain of the mRNA export receptor Nxf1, allowing the stable association of Nxf1 with mRNA which subsequently leads to transport of the mRNA to the cytoplasm [2]. Thus TREX acts to license mRNA export, informing the cell when an mRNA is processed and suitable for export. TREX is a multisubunit complex whose assembly requires ATP [3]. Four subunits of TREX are known to make contact with Nxf1, these are Alyref, Thoc5, Hpr1 and Chtop [4] [5]. Chtop and Thoc5 both bind to the same domain of Nxf1 and both cooperate with Alyref to enhance the RNA binding activity of Nxf1. However, Nxf1, Chtop and Alyref all exist in a single complex *in vivo*, indicating that the TREX complex is likely to undergo significant structural rearrangements during maturation of the mRNP. Both Chtop and Alyref are regulated by arginine methylation [5] [6] and in the case of Chtop, the methylation of arginines allows it to bind Nxf1.

Although many biochemical approaches have been used to study the TREX complex function in gene expression, the sites within the nucleus where TREX recruits Nxf1 to mRNA are still unclear. The nuclear speckles are thought to be storage, assembly and modification sites for splicing and export factors [7] [8] and a number of TREX subunits have been mapped to nuclear speckles in fixed cells [5] [9] [10]. mRNA splicing has been shown to take place in the vicinity of the perichromatin fibrils which surround nuclear speckle domains [11] [12] and several studies have shown that splicing factors can be recruited from speckles to actively transcribed genes at the periphery of a speckle [13]–[15]. However, recent work has shown that post-transcriptional splicing occurs within the nuclear speckle and that release of mRNA from nuclear speckles and subsequent export to the cytoplasm requires the TREX complex [16], [17]. Despite all these studies, the site within the nucleus where Nxf1 assembles with the TREX complex remains unknown.

We have used two fluorescent imaging techniques to study the interaction of TREX components with Nxf1, sensitized emission FRET and FLIM-FRET. Both techniques have the advantage that they rely on molecules being within 1–10 nm and therefore are likely to report genuine protein-protein interactions [18]. In sensitized emission FRET the donor is excited with light of a suitable wavelength and fluorescence is measured in the acceptor channel. However, the signal in the acceptor channel does not only arise from FRET. The light used to excite the donor also causes some excitation of the acceptor due to spectral overlap between the donors and acceptors commonly used. To alleviate this problem images are also collected for donor only and acceptor only, excited with the same wavelengths used to excite the donor when measuring FRET. These control images are used to substract the fluorescence caused by spectral bleedthrough, which should in principal just leave the FRET signal, though correct acquisition of control images and substraction is vital to ensure an accurate FRET image is produced. In FLIM-FRET, the time it takes for a fluorophore to become excited and then return to ground state is measured. The lifetime of the fluorescence of the donor molecule decreases when the donor molecule is engaged in FRET with an acceptor molecule, therefore the fluorescence lifetime of the donor molecule provides a read out of molecules engaged in FRET and are likely to be interacting. Since FLIM-FRET only measured donor fluorescence and is not subject to potential problems associated with spectral crosstalk it provides a robust readout of protein interactions in living cells. In addition, FLIM-FRET allows quantification of the number of interacting molecules at specific sites within the cell with nanometer resolution [19]. By using both FRET techniques we aimed to produce an accurate and coherent view of the interactions between mRNA export factors in human cells. Our results provide the first intranuclear spatial map of the assembly of the export competent mRNP. We show that TREX assembly with Nxf1 predominantly occurs outside nuclear speckles, despite a large proportion of TREX subunits residing within nuclear speckles at steady state.

## Materials and Methods

### Plasmids, antibodies and cell cultures

The full-length Nxf1, Chtop, Alyref, the C-terminus of Nxf1 (the 372–619 fragment) or deletion mutants of Chtop (fragments corresponding to amino acids 1–87 and 92–213) were PCR amplified and cloned into pECFP-N1, pEYFP-N1 or pEGFP-N1 vectors. Human HeLa cells were grown on 35 mm glass bottom dishes with DMEM (Invitrogen) supplemented with 10% Fetal Calf Serum and 100 U/ml of penicillin and streptomycin (Invitrogen) and incubated at 37 °C with 5% CO_2_. Cells were transfected using Turbofect (Fermentas). The Nxf1 and Hpr1 antibodies were from Abcam. The Alyref monoclonal antibody (11G5) was from Sigma. The Thoc5 and Chtop (KT64) antibodies were described [10] [20]. The Thoc5 antibody was described previously [10]. For inhibition of transcriptional activity, cells were treated for 2 hours with 10 µg/ml actinomycin D (Sigma-Aldrich) prior to imaging analysis.

### Immunoprecipitation

Cells were transfected with a construct expressing the target protein or mock transfected for 48 hours, each dish was lysed in 1 mL of IP lysis buffer (50 mM HEPES pH 7.5, 100 mM NaCl, 1 mM EDTA, 1 mM DTT, 0.5% Triton X-100, 10% Glycerol) containing protease inhibitors and 10 µg/mL RNase A. The supernatants of cell extracts were incubated for 1 hour with 30 µL Protein G-Sepharose beads in IP lysis buffer supplemented with 1% BSA. The anti-GFP monoclonal antibody (Roche) was bound to 30 µL Protein G-Sepharose beads for 1 hour prior to immunoprecipitation. The beads were then washed with 1 mL IP lysis buffer three times. The bound proteins were finally eluted from the Protein G-Sepharose with 50 µL of buffer (0.2 M glycine pH 2.8, 1 mM EDTA), and analysed by SDS-PAGE and Western blotting with the indicated antibodies.

### Immunofluorescence microscopy

HeLa cells were fixed in 4% paraformaldehyde in PBS for 15 mins and permeabilized with 0.1% Triton X-100 in PBS for 10 mins before immunostaining. After blocking with 2% bovine serum albumin (BSA) in PBS for 1 hour, cells were incubated with the primary antibodies for 1 hour at room temperature. Then cells were washed in PBS three times and incubated with the fluorescently labeled secondary antibody (AlexaFluor 488 or 555) for 1 hour. Cells were washed in PBS three times and nuclei were counterstained with 4,6-diamidino-2-phenylindole (DAPI) for microscopy analysis.

### Confocal microscopy and fluorescence resonance energy transfer analysis

Cell images were collected using a Zeiss LSM 510 META microscope equipped with a Zeiss Plan-Apochromat 63× NA 1.4 oil immersion DIC lens. For Z-stack confocal images a micrometer step size was used for the z-scan. *In situ* characterisation of fluorescence emission spectra was performed using the Zeiss META detection module with a 458 nm laser excitation. For sensitized emission FRET, a 30 mW Argon laser line 458 nm was used for ECFP (donor) and FRET excitation and laser line 514 nm for EYFP (acceptor) excitation. To effectively reduce background noise, emission fluorescence images of ECFP, EYFP, and FRET pairs were acquired with band pass filter BP 470–500, long pass filter LP530, and long pass filter LP530, respectively.

To measure the normalized FRET (NFRET) value, all three emission images from cells expressing FRET pairs were collected and processed using the Image J (National Institutes of Health) FRET plug-in based on this equation: 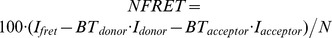. Cells expressing donor alone or acceptor alone were acquired to measure spectral bleed through coefficients BT_donor_ or BT_acceptor_. N was determined by the square root of the product of donor and acceptor intensities. For quantitative analysis, mean NFRET values were determined by defining regions of interest (ROIs) for the whole nucleus. Data were presented as mean value ± SD of independent experiments and *P*-values were obtained using the Student's *t*-test. *P*<0.05 was considered as statistically significant. An NFRET value of >5% was considered a significant protein-protein interaction.

### TCSPC fluorescence lifetime imaging microscopy

FLIM was performed using an upright multiphoton Zeiss LSM510 NLO laser scanning microscope with a 60× NA1.0 water immersion lens. A Coherent Chameleon Verdi-pumped ultrafast tunable (690–1040 nm) laser was used for multiphoton excitation by pumping a mode-locked Ti:Sapphire laser to produce sub-200 femtosecond duration pulses at a 90 MHz repetition rate. Fluorescence lifetimes were acquired by the high-speed Hamamatsu 5783P detector and each photon was delivered to B&H SPCM/SPC-830 TCSPC imaging module board. To measure the lifetime of fluorophore, ECFP fusion protein was excited by multiphoton laser at 850 nm and emission fluorescence was collected to bandpass filter 435±50 nm. A mean photon count rate of each image was monitored at the order 10^4^∼10^6^ photons per second and acquisition time was over 90 seconds. Time-correlated single-photon counting (TCSPC) measurement relies on the fluorescence decay histogram generated from accumulated photon counts at different times after the laser excitation pulse. The recorded data was analyzed using B&H SPCImage software. Measurements of FRET based on the analysis of the fluorescence lifetime of the donor by FLIM approach can resolve the FRET efficiency and the FRET population (concentration of FRET species) when analyzed using bi-exponential decays model. In the analysis of FLIM-FRET data, a bi-exponential decay model (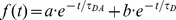) was fitted in ECFP fusion proteins and FRET pair specimens. The displayed X^2^ value was optimized to close to one as possible to achieve a best fitting model. We obtain information about the lifetimes of two populations of molecules, τ_DA_ is the mean fluorescence lifetime of the donor in the presence of the acceptor and τ_D_ is the mean fluorescence lifetime of the donor expressed in the absence of acceptor as well as two decay components a and b. By fixing the non-interacting proteins lifetime τ_D_ using data from control experiments (in the absence of FRET) and by assuming invariance in the efficiency of interaction τ_DA_, the population of FRET species can be estimated. From this model, the FRET efficiency was measured by the equation: 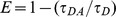. For quantitative analysis, the mean value for FRET efficiency was determined by defining regions of interest (ROIs) for the whole nucleus. Data were presented as means ± SD of independent experiments and *P*-values were obtained using Student's *t*-test. *P*<0.05 was considered as statistically significant. A FRET efficiency of >5% is considered a significant protein-protein interaction.

### Fluorescence recovery after photobleaching

HeLa cells expressing EGFP fusion proteins were grown in glass bottom dishes. FRAP experiments were performed using a Zeiss LSM 510 META microscope equipped with a Zeiss Plan-Apochromat 63× NA 1.4 oil immersion DIC lens. A small circular area within the nuclear speckle or nucleoplasm was bleached using 100% 488 nm laser power. The four images were acquired before bleaching with 1.5∼2.5% 488 nm laser power. A series of 76 post-bleached images for the Chtop-GFP in the nuclear speckles were captured at intervals of 15 s. A series of 76 post-bleached images for Chtop-GFP or mutant Chtop-GFP in the nucleoplasm were collected at interval of 5 s and 98.9 ms. A series of 76 post-bleached images for Alyref-GFP or Nxf1-GFP in the nuclear speckles or nucleoplasm were captured at intervals of 58.8 ms. Fluorescent intensities of the bleached area collected at different series of time were normalized to pre-bleached fluorescent intensity. For the measurement of half-time of recovery (τ*_1/2_*) and immobile fraction, we used the mathematical equation: 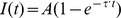, whereas A indicates the mobile fraction, I(t) is the fluorescent intensity at time (t), and τ is the lifetime of recovery to fit the frap curve and meanwhile the value of the immediate post-bleached intensity as 0 and the pre-bleached intensity as 1 were carried out.

## Results

### Subnuclear localization of mRNA export proteins

**Figure 1:**
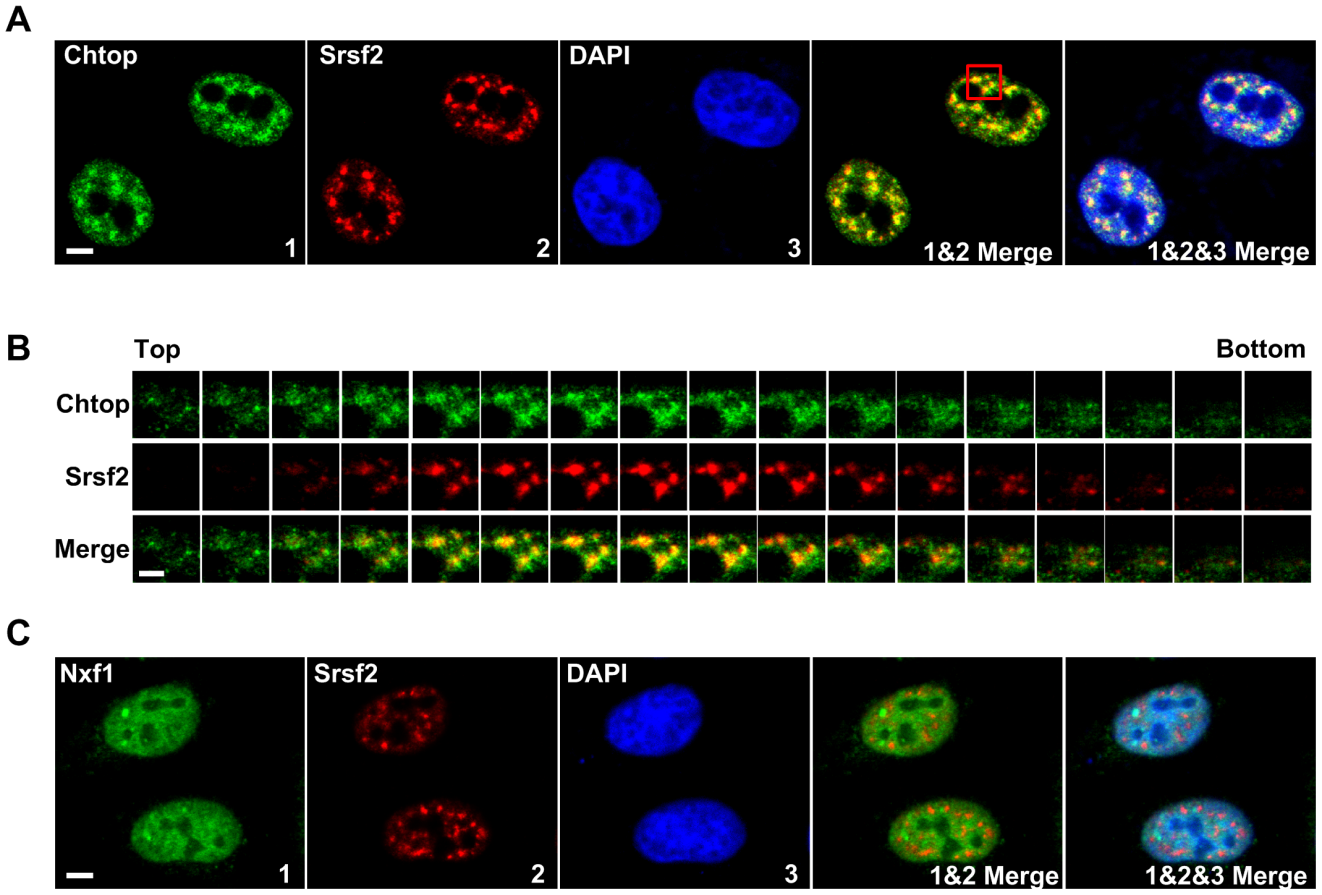
Chtop colocalizes with Srsf2 in the nuclear speckles. (A) Representative images of HeLa cells stained with anti-Chtop antibody (green), anti-Srsf2 antibody (red), and DAPI (blue) to visualize intranuclear localization of Chtop and Srsf2. Scale bar, 5 µm. (B) Z-stack images represents red box inset in A. Scale bar, 2 µm. (C) Representative images of HeLa cells stained with anti-Nxf1 antibody (green), anti-Srsf2 antibody (red), and DAPI (blue) are shown to visualize intranuclear localization of Nxf1 and Srsf2. Scale bar, 5 µm.

To investigate the subnuclear distribution of mRNA export factors in cells we performed immunofluorescence microscopy. The Srsf2 (SC35) antibody was used to label the nuclear speckles which coincided with areas of the nucleus that stained poorly with the DNA stain DAPI (Figure 1A). The cells were also stained for the TREX subunit Chtop, which was concentrated in and around nuclear speckle domains (Figure 1A). Close examination of z-stack sections revealed that whilst the majority of Chtop colocalised with Srsf2 in nuclear speckles, a significant proportion of Chtop localised to the region immediately surrounding the speckle domains (Figure 1B). We also examined the localization of endogenous Nxf1 in Hela cells and found it had a diffuse nuclear staining with no obvious concentration in nuclear speckle domains (Figure 1C).

### Detection of the Chtop:Nxf1 interaction in living cells

**Figure 2:**
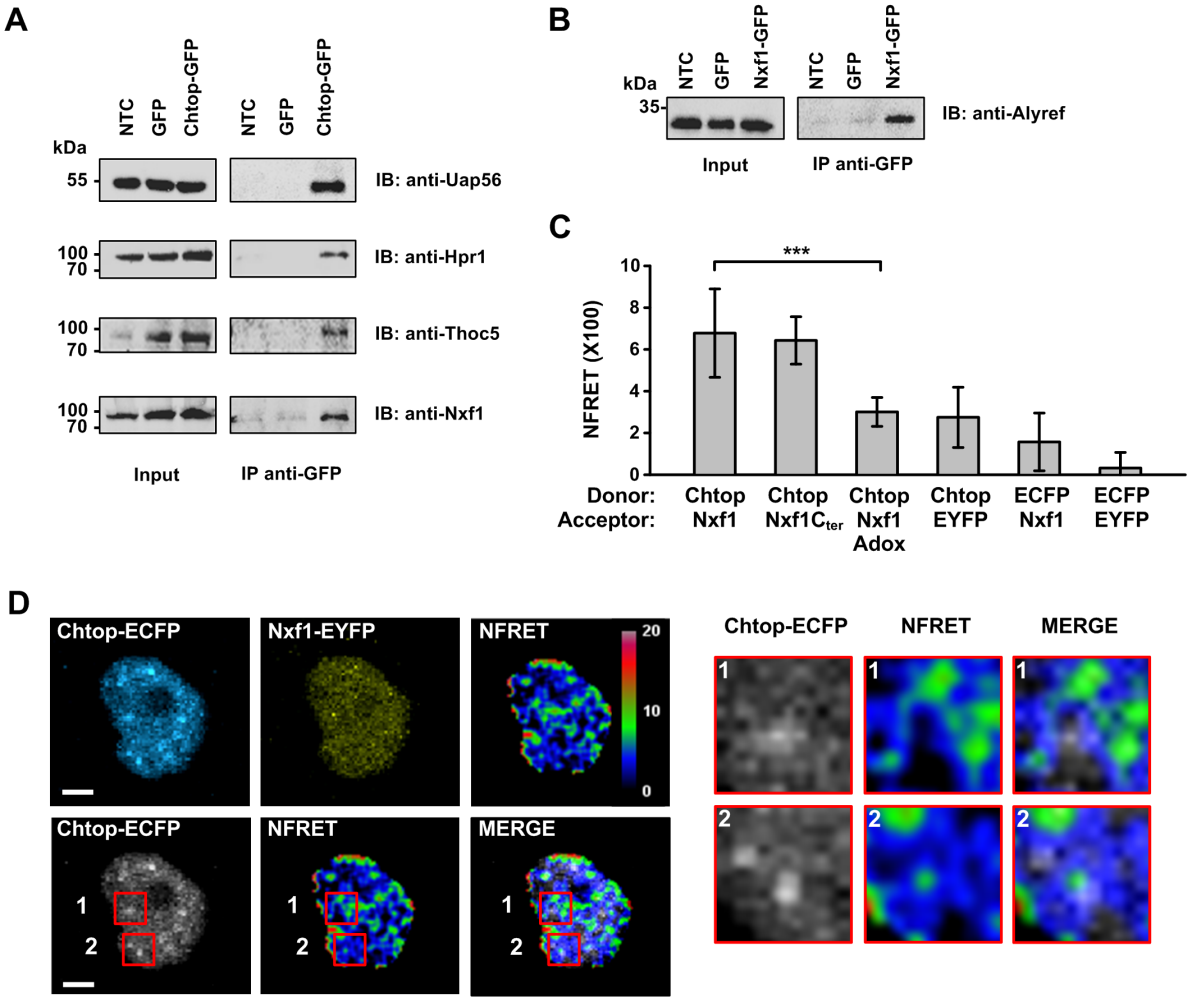
TREX complex associates with Nxf1 in the vicinity of nuclear speckles. (A and B) 293T cells transiently expressing Chtop-GFP, Nxf1-GFP, GFP or mock transfected were immunoprecipitated with anti-GFP antibody and detected by Western blot with the indicated antibodies. (C) Quantitative NFRET values were analyzed by sensitized emission FRET. FRET pairs were displayed from left to right in the panel: donor Chtop-ECFP and acceptors Nxf1-EYFP, Nxf1C-terminus-EYFP, Nxf1-EYFP, EYFP; control pairs donor ECFP and acceptors Nxf1-EYFP, EYFP. Data presented as mean ± SD for n = 22–25 cells, ***P<0.001. (D) The upper panel showed representative images of live HeLa cells coexpressing Chtop-ECFP and Nxf1-EYFP. The NFRET image is shown in color-code format with percentage value ranges. The lower panel representing the merged image was combined from Chtop-ECFP and NFRET. Magnified images were displayed as red box insets. Scale bar, 5 µm.

To investigate the localization of Chtop in live cells we firstly investigated whether Chtop tagged with a fluorescent protein was capable of interacting with other mRNA export factors. Chtop-GFP was found to co-immunoprecipitate with multiple TREX subunits and Nxf1, indicating that the fluorescent protein tag does not prevent its assembly with these binding partners (Figure 2A). Similarly, tagging Nxf1 with GFP did not prevent its association with mRNA export factors since it co-immunoprecipitated with Alyref (Figure 2B). To directly examine the interaction of Chtop and Nxf1, we performed sensitized emission FRET in live HeLa cells using Chtop-ECFP as donor and Nxf1-EYFP as acceptor. The normalized FRET (NFRET) measured from the entire nucleus of a cell in control donor:acceptor pairs of ECFP:Nxf1-EYFP, Chtop-ECFP:EYFP and ECFP:EYFP were averaged (Figure 2C). For all these control samples the averaged NFRET values were below 5.0, indicating this is a baseline value for non-specific NFRET in these conditions. In contrast the Chtop-ECFP:Nxf1-EYFP pair showed an average NFRET signal of 6.78, which is significantly higher than the background (Figure 2C). Since Chtop interacts with the NTF2-like (NTF2L) domain of Nxf1, we also examined this interaction using a C-terminal fragment of Nxf1 encompassing the NTF2L domain and found this also gave an NFRET signal significantly higher than the background. The interaction of Chtop with the NTF2L domain of Nxf1 requires methylation of Chtop [5]. Therefore we also examined the NFRET signal in cells expressing Chtop-ECFP:Nxf1-EYFP in the presence of the methylation inhibitor Adox and found that the NFRET signal was reduced to background levels. Together these data indicate that the FRET signal observed between Chtop-ECFP:Nxf1-EYFP is specific and correlates with previous biochemical experiments used to analyse this interaction [5]. To spatially map the interaction between Chtop-ECFP and Nxf1-EYFP in the nucleus, NFRET images were displayed in a color-code format (Figure 1D). Strikingly, the pattern of NFRET signal differed markedly from the localization observed for Chtop-ECFP and Nxf1-EYFP individually. There was a strong NFRET signal at the nuclear periphery together with strong patches of intranuclear signal. Since the most intense Chtop staining in the nucleus in fixed cells corresponds with Srsf2 in the nuclear speckles (Figure 1B) we also overlayed the NFRET signal with the Chtop-ECFP signal (Figure 2D lower and right panels). This overlay showed that the strong intranuclear patches of NFRET signal do not signficantly overlap with nuclear speckle regions, but some interaction sites lie in close proximity to speckles. Together these data suggest that the major sites for interaction of Nxf1 and Chtop are in close proximity to nuclear speckles. There are additional intranuclear sites for strong Nxf1:Chtop interactions mapped by NFRET, not obviously associated with nuclear speckles and some of these may correspond to Chtop and Nxf1 assembled in the mRNP in transit to the nuclear pore. The interaction sites at the nuclear rim may correspond to mRNPs docked at the nuclear pore, in the process of or awaiting translocation through the nuclear pore.

**Figure 3:**
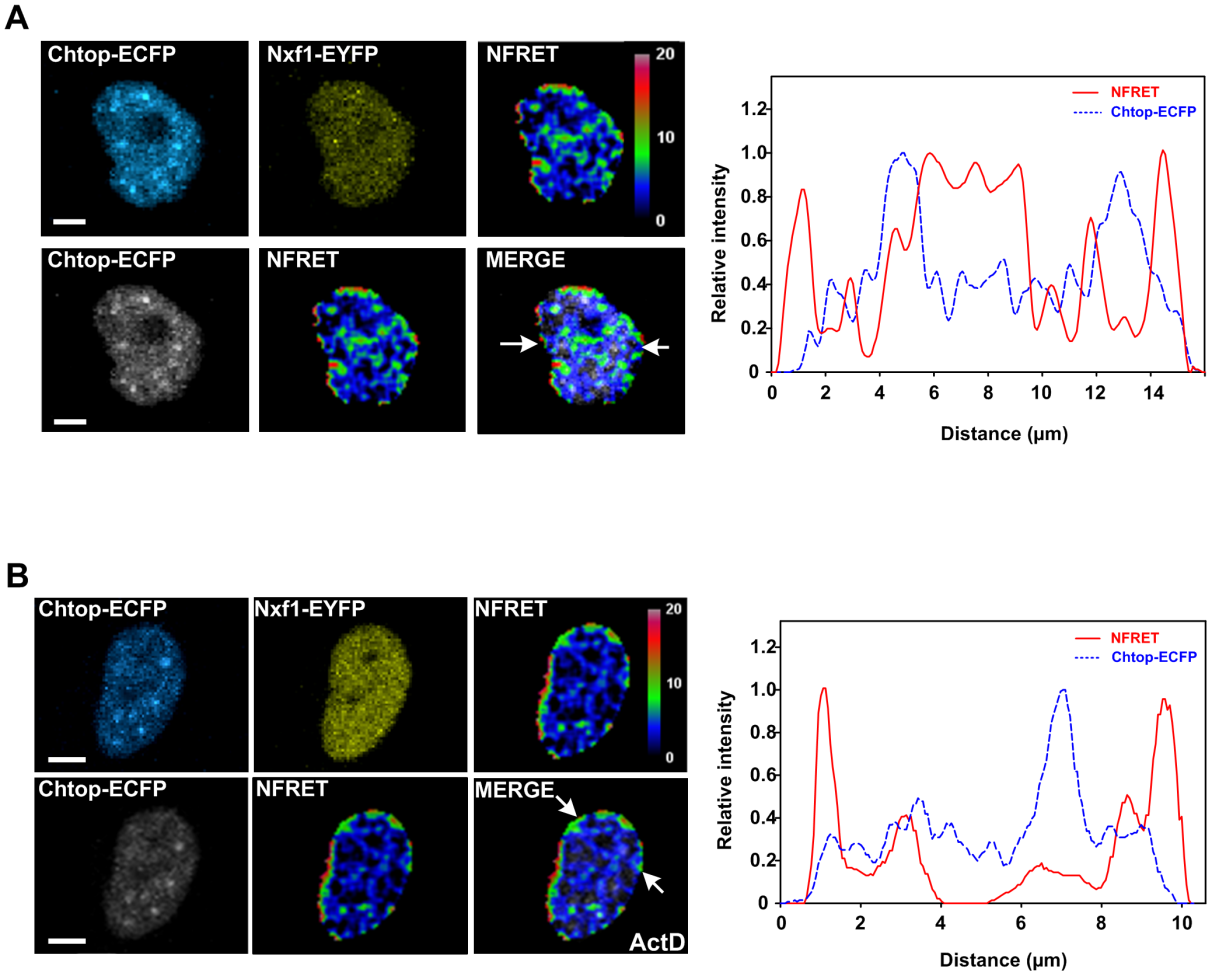
The location of Nxf1:Chtop interactions are altered following transcription inhibition. Representative NFRET images of HeLa cells coexpressing Chtop-ECFP and Nxf1-EYFP in the absence (A) or presence of actinomycin D treatment (B). The arrows indicate the position of the line scan which was used to quantitate the Chtop-ECFP and NFRET intensity across cells and the results are shown in the graphs on the right hand side. Scale bar, 5 µm.

We also assessed the importance of ongoing transcription for the Chtop-ECFP:Nxf1-EYFP interaction (Figure 3A, B). The NFRET signal was quantitated across cells and we found that there was a significant reduction in the intranuclear NFRET signal when cells were treated with actinomycin D. In contrast, the NFRET signal at the nuclear periphery persisted. These data suggest that the intranuclear interaction between Chtop and Nxf1 requires ongoing transcription yet the interaction at the nuclear periphery does not.

### Spatially mapping the interaction between Chtop and Nxf1 by FLIM-FRET

**Figure 4:**
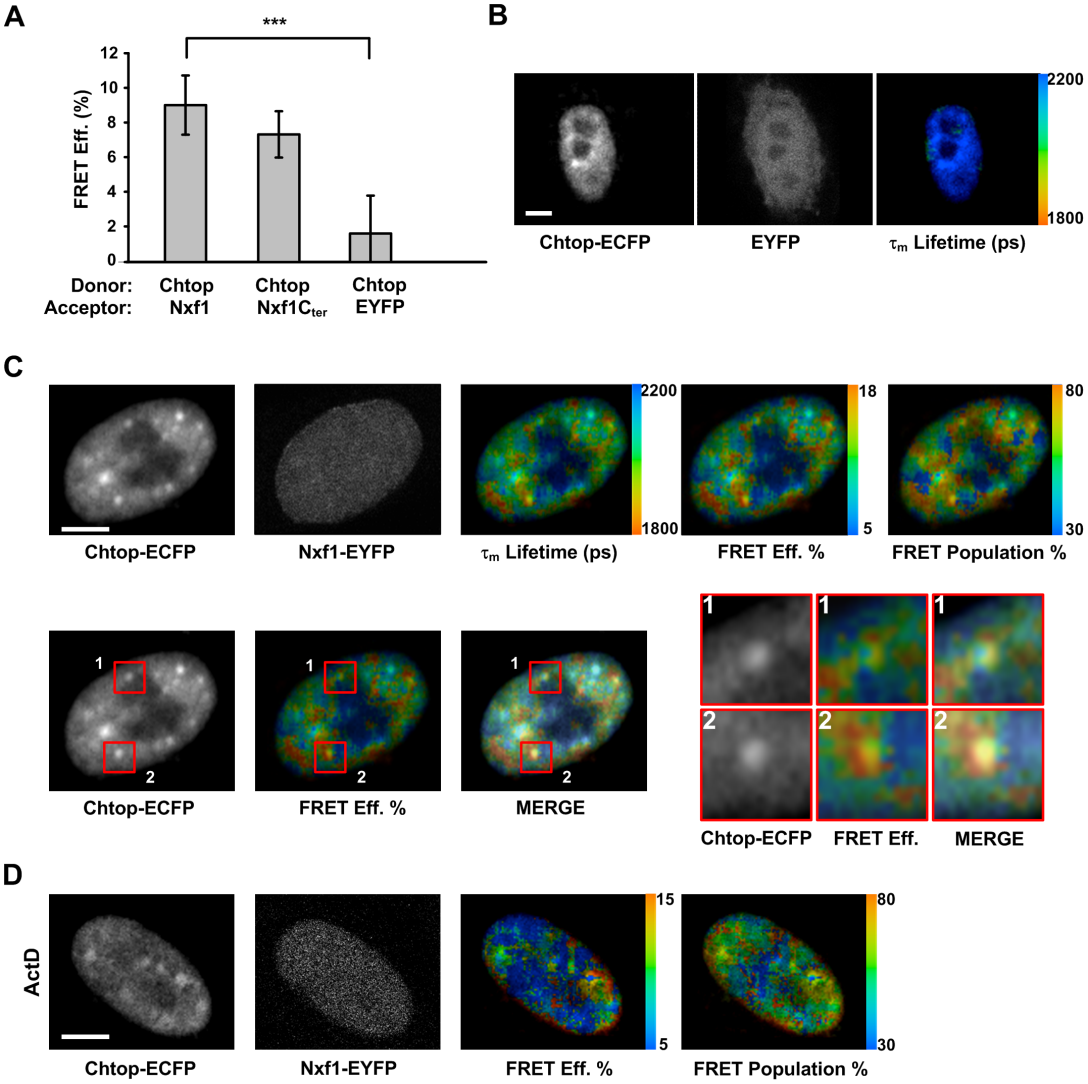
Topological relationship between Chtop and Nxf1 analysed by FLIM-FRET. (A) HeLa cells were transfected with both donor Chtop-ECFP and indicated acceptors Nxf1-EYFP, Nxf1C-terminus-EYFP and EYFP. Bar graph of FRET efficiency was analyzed by FLIM-FRET. Data presented as mean ± SD for n = 15–31 cells, ***P<0.001. (B) Representative images of HeLa cells co-expressing Chtop-ECFP and EYFP. The image representing mean fluorescence lifetime (picoseconds) was shown in continuous pseudo-colors with time value range. (C) Representative images of HeLa cells coexpressing Chtop-ECFP and Nxf1-EYFP. FRET efficiency and FRET population (% of interacting donor molecules) were shown in continuous pseudo-colors with percentage value ranges. (D) Effect of actinomycin D treatment on the interaction between Chtop-ECFP and Nxf1-EYFP. Scale bar, 5 µm.

To further characterise the Chtop:Nxf1 interaction *in vivo* we performed FLIM-FRET analysis. When Chtop-ECFP was expressed with EYFP a background average FRET efficiency of 1.59% was observed, whereas when cells expressed both Chtop-ECFP and Nxf1-EYFP the FRET efficiency rose to 9.00% (Figure 4A). A similar robust interaction was detected for Chtop-ECFP together with a construct expressing the C-terminal half of Nxf1 fused to EYFP. To map the intracellular distribution of the FLIM-FRET signal, images from HeLa cells co-expressing Chtop-ECFP and Nxf1-EYFP were mapped with continuous pseudocolors in each pixel to show mean fluorescence lifetime, the percentage of FRET efficiency and FRET population. To establish the background FLIM-FRET signal we analysed Chtop-ECFP co-expressed with EYFP and observed relatively long fluorescence lifetimes throughout the nucleus, thus providing a baseline for non-specific interactions (Figure 4B). In contrast, Chtop-ECFP co-expressed with Nxf1-EYFP gave an image with much lower fluorescence lifetimes within the nucleus, indicative of a specific interaction (Figure 4C). The steady state localisation of Chtop overlaps with nuclear speckles and therefore the Chtop-ECFP signal provides a guide as to the location of the nuclear speckles. Strikingly, when the FLIM-FRET signal for Chtop-ECFP:Nxf1-EYFP was overlayed with the Chtop-ECFP signal it became apparent that the main sites for interaction between Chtop-ECFP and Nxf1-EYFP were found in close proximity to the speckle regions, together with additional intranuclear regions not directly associated with speckles. Within the nuclear speckles, there was still evidence of an interaction above background levels but at a much lower level than that seen on the periphery of speckles and at other intranuclear sites. With actinomycin D treatment, the FLIM-FRET efficiency signals between Chtop-ECFP and Nxf1-EYFP were reduced within the nucleus (Figure 4D) but strong interaction sites were visible around the nuclear periphery as observed earlier (Figure 3B).

### Alyref interacts with Nxf1 in living cells

**Figure 5:**
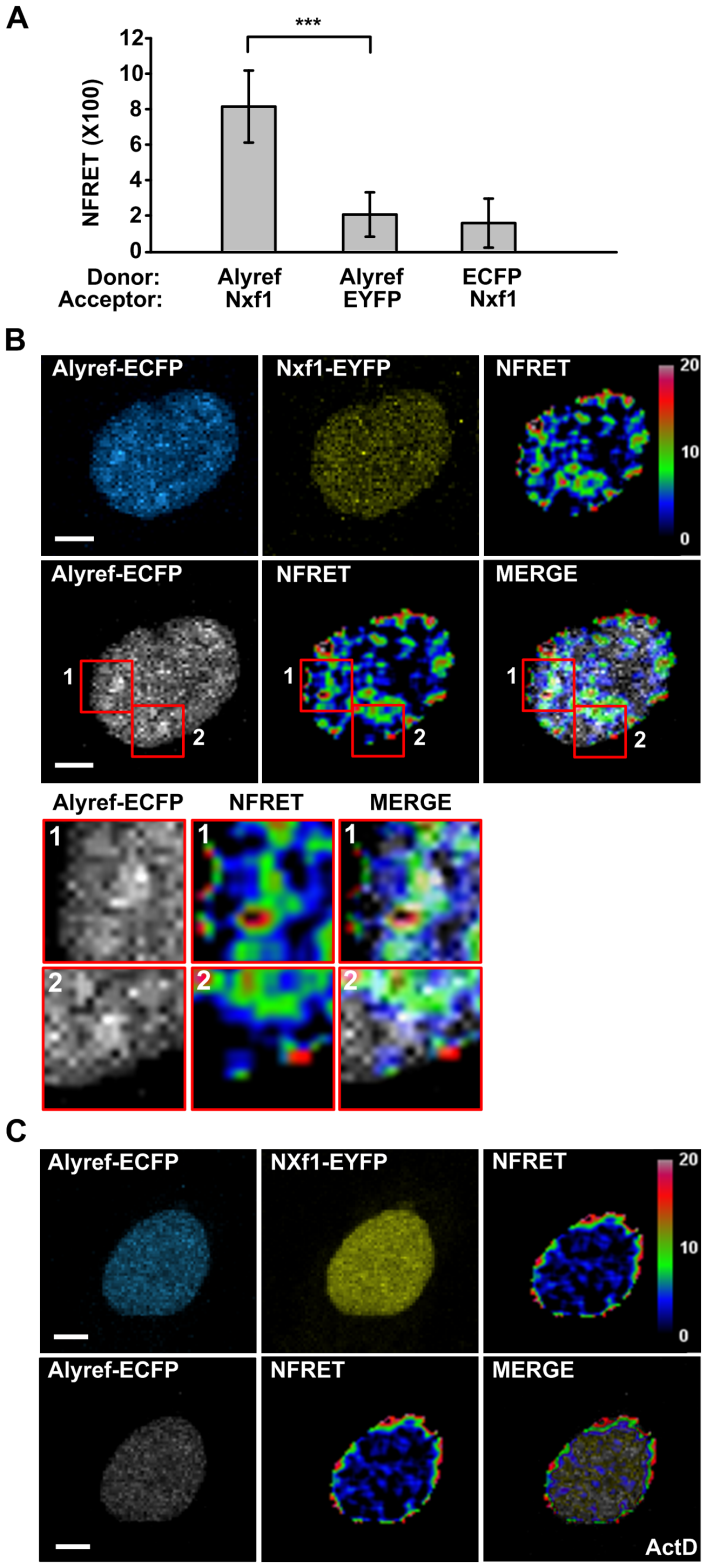
Analysis of the Nxf1:Alyref interaction in living HeLa cells. (A) Quantitative analysis of NFRET values for different pairs of donor and acceptor in live HeLa cells by sensitized emission FRET (mean ± SD for n = 22–32 cells, ***P<0.001). (B) Representative NFRET images of HeLa cells coexpressing Alyref-ECFP and Nxf1-EYFP. (C) Effect of actinomycin D treatment on the interaction between Alyref-ECFP and Nxf1-EYFP examined by NFRET. Scale bar, 5 µm.

To establish whether the pattern of interaction observed between Nxf1 and Chtop was a more general property of other mRNA export factors which interact with Nxf1, we also monitored the Alyref-ECFP:Nxf1-EYFP interaction in living cells. Initially we analysed the overall NFRET-signal observed in the nucleus, averaged across multiple cells. When Alyref-ECFP co-expressed with EYFP and ECFP co-expressed with Nxf1-EYFP gave background NFRET signals of 2.06 and 1.57 respectively. In contrast, Alyref-ECFP:Nxf1-EYFP gave a significantly higher NFRET value of 8.14, indicating a robust interaction (Figure 5A).

**Figure 6:**
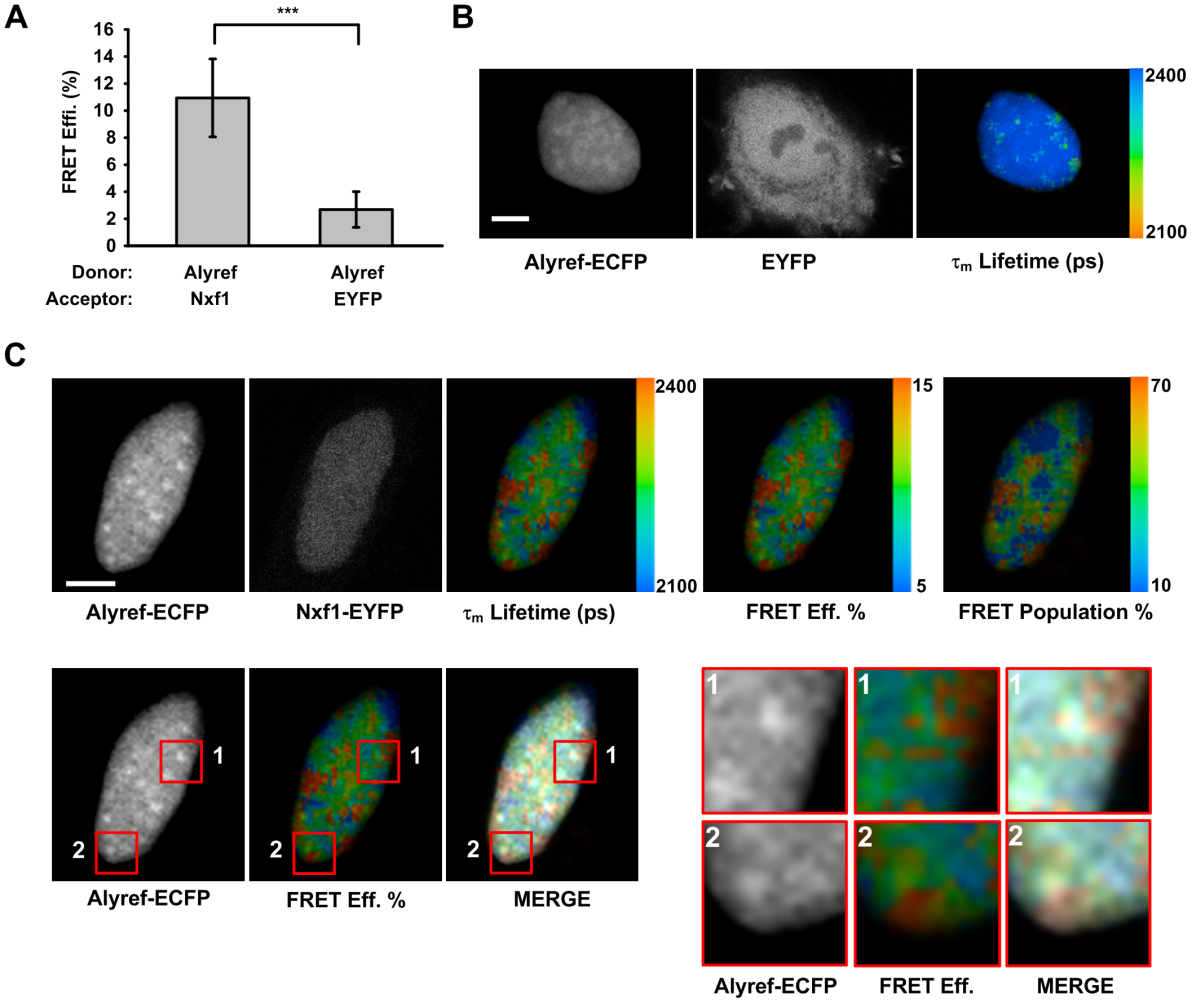
The spatial interactions of Nxf1 and Alyref in single cells by FLIM-FRET. (A) Bar graph of FLIM-FRET efficiency of donor Alyref-ECFP with acceptor Nxf1-EYFP or EYFP (mean ± SD for n = 18–27 cells, ***P<0.001). (B) Representative FLIM-FRET images of HeLa cells coexpressing Alyref-ECFP and EYFP. (C) Representative FLIM-FRET images of HeLa cells transfected with both Alyref-ECFP and Nxf1-EYFP. Scale bars, 5 µm.

We next analysed the distribution of NFRET signals for Alyref-ECFP coexpressed with Nxf1-EYFP. Since Alyref localizes with nuclear speckles at steady state [9], we overlayed the NFRET and Alyref-ECFP images (Figure 5B). This analysis revealed that the major sites of interaction of Alyref-ECFP and Nxf1-EYFP did not coincide with the nuclear speckle regions directly although they were closely associated (Figure 5B, lower panels). We also observed that, actinomycin D treatment reduced the intranuclear NFRET signal for Alyref-ECFP and Nxf1-EYFP, yet a strong signal persisted at the nuclear periphery. This striking alteration in the sites of interactions mirrors that observed between Chtop-ECFP and Nxf1-EYFP (Figure 3B). Further analysis of the Alyref-ECFP:Nxf1-EYFP interaction using FLIM-FRET (Figures 6A–C) confirmed the specific association of Alyref and Nxf1 *in vivo*. The major sites of association between Alyref-ECFP and Nxf1-EYFP did not correspond to the speckle regions where Alyref is found at steady state [9] and Figure 6C. Instead, major interaction sites were found in close proximity to the speckles within the nucleus (Figure 6C lower panels). However, FLIM-FRET detected interactions between Nxf1 and Alyref even within speckle regions, though at much lower levels, consistent with the FLIM-FRET analysis of the Chtop-ECFP:Nxf1-EYFP interaction.

### Intermolecular interactions between mRNA export factors Chtop and Alyref *in vivo*

**Figure 7:**
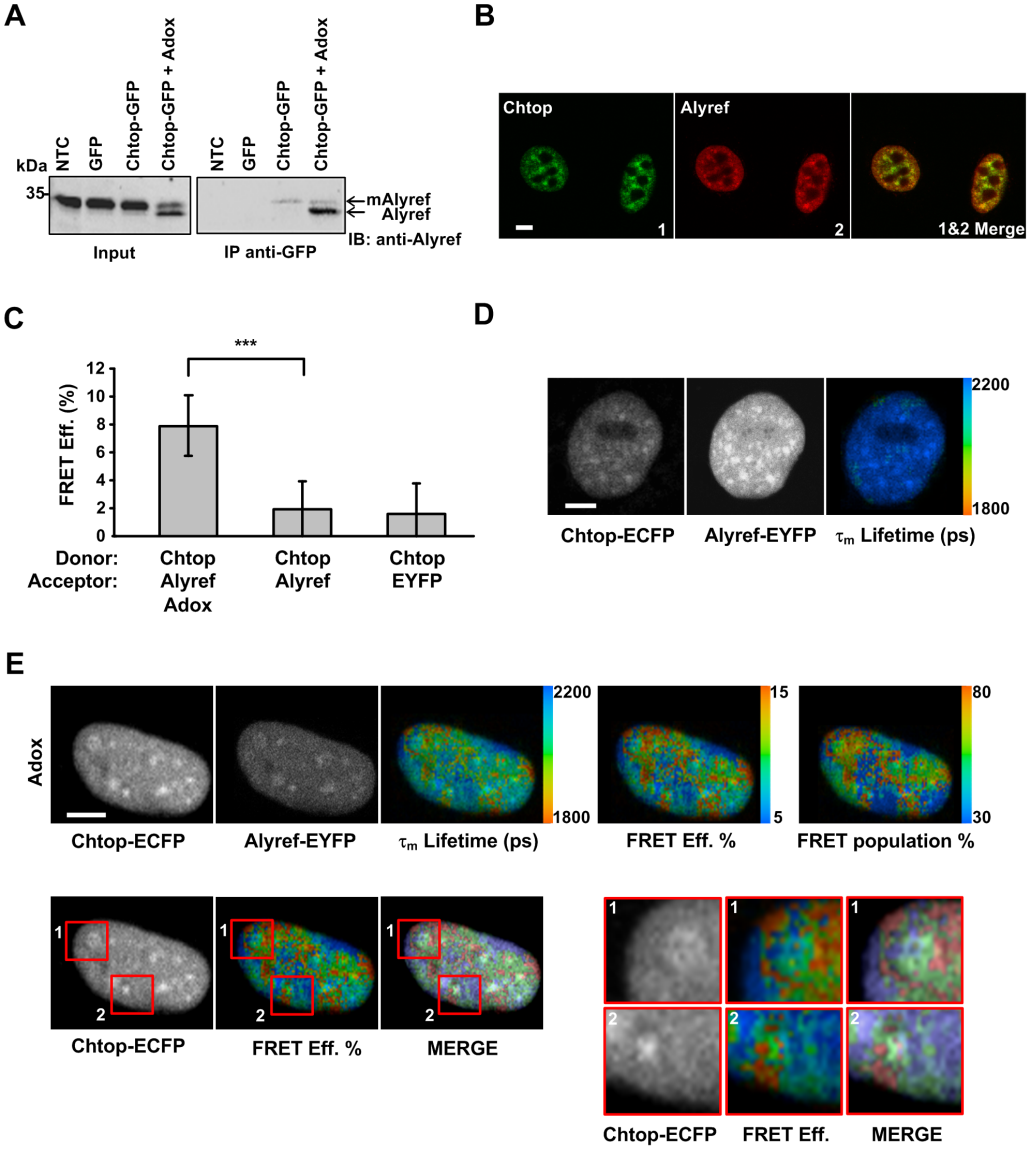
Spatial interactions of Chtop and Alyref in the vicinity of nuclear speckle. (A) cells extracts from 293T cells transfected with Chtop-GFP, GFP or mock transfected were immunoprecipitated with anti-GFP antibody and detected by Western blot with anti-Alyref antibody. (B) The fixed HeLa cells were stained with anti-Chtop antibody (green) and anti-Alyref antibody (red). (C) Quantitative FLIM-FRET efficiency of Chtop-ECFP and Alyref-EYFP pair in the presence or absence of Adox treatment (mean ± SD for n = 17–25 cells, ***P<0.001). (D) Representative FLIM-FRET images of HeLa cells coexpressing Chtop-ECFP and Alyref-EYFP (E) Effect of Adox treatment on the interaction between Chtop-ECFP and Alyref-EYFP. NTC, nontransfected cells; mAlyref, methylated Alyref; Scale bars, 5 µm

To investigate where TREX assembly occurs in the cell we investigated where two TREX subunits Chtop and Alyref interact. Chtop exists in both methylated and unmethylated forms within the nucleus. Its methylation state can regulate the intermolecular interactions with Alyref, Nxf1 and RNA, with unmethylated Chtop selectively binding Alyref and methylated Chtop binding Nxf1 [5]. We established that Chtop tagged with a fluorescent protein retained the same binding characteristics with Alyref using co-immunoprecipitation (Figure 7A). Following treatment with Adox we observed a characteristic shift to a higher mobility form of Alyref in SDS-PAGE which corresponds to the hypomethyalted form (Figure 7A), indicating Adox treatment was successfully blocking protein methylation. Following immunoprecipitation with an antibody to GFP we found that Chtop-GFP preferentially immunoprecipitated with Alyref when cells were treated with Adox (Figure 7A, right panel), indicating the interaction between these proteins preferentially occurs when they are hypomethylated.

We next investigated the steady state localization of both Chtop and Alyref and found that they showed substantial colocalisation (Figure 7B) consistent with their individual colocalisation with nuclear speckles (Figure 1A and [9]). We examined where Chtop and Alyref interactions occur in living cells using FLIM-FRET (Figures 7C,D,E). In the absence of Adox only background signals were observed for Chtop-ECFP:Alyref-EYFP (Figures 7C,D). This suggests that unmethylated Chtop only exists very transiently in cells normally. Following Adox treatment, strong intranuclear interactions were observed by FLIM-FRET (Figure 7E). The FRET population and FRET efficiency maps showed substantial similarities and when the steady state localization of Chtop-ECFP was compared with the FRET efficiency map it was clear that the major Chtop-ECFP and Alyref-EYFP interactions occur in regions adjacent to nuclear speckles and at additional intranuclear sites. However the FRET efficiencies within regions corresponding to nuclear speckles were above background, though substantially lower than those seen on the periphery of speckles, indicating Chtop and Alyref also associate within nuclear speckles.

### Nuclear dynamics of mRNA export proteins

**Figure 8:**
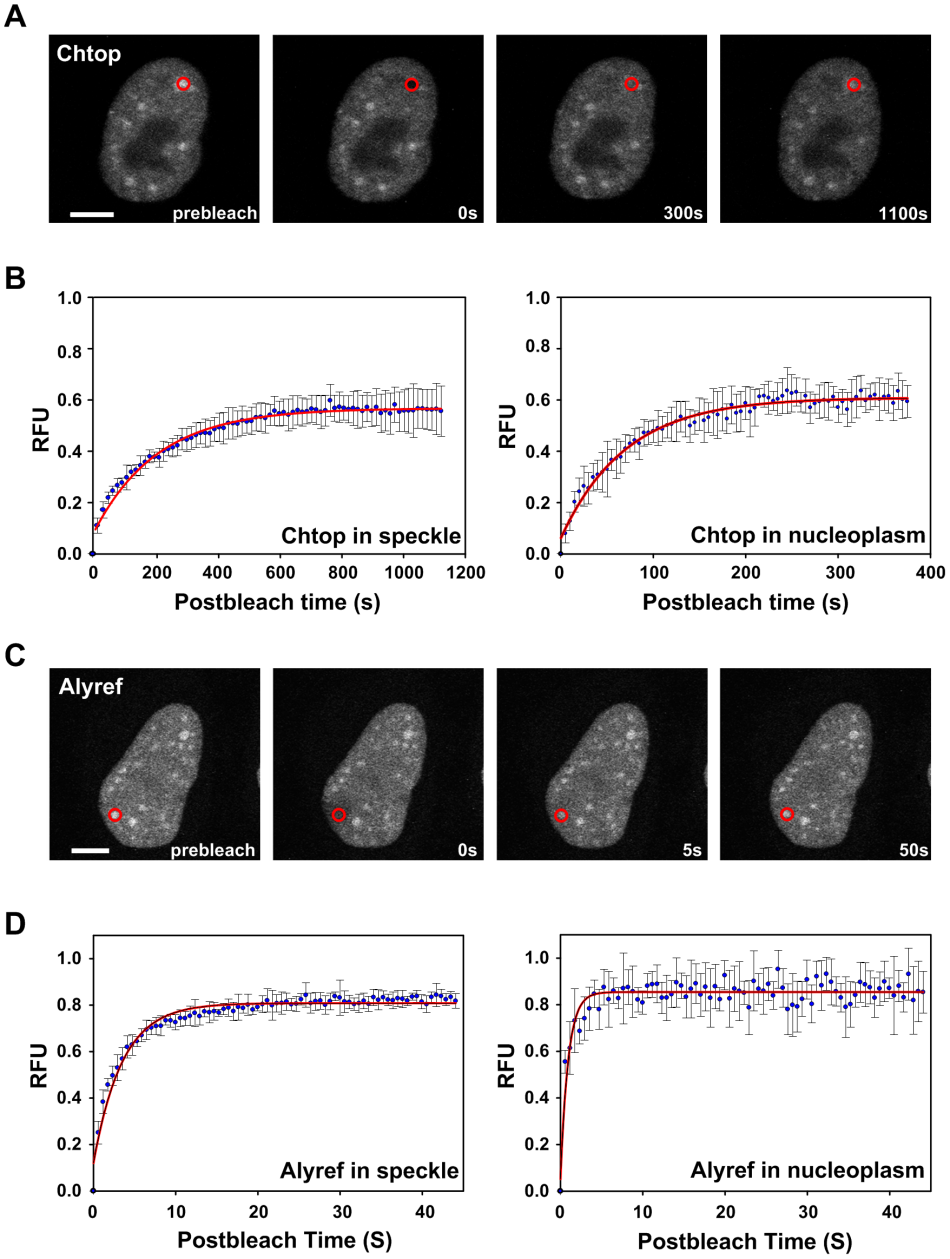
Nuclear dynamics of mRNA export proteins were measured by FRAP. (A) Representative images illustrating fluorescence recovery of Chtop-GFP after photobleaching in nuclear speckle (red circle) of live HeLa cells. (B) The plots of time-dependent normalized fluorescence recovery of Chtop-GFP in the bleached speckles and nucleoplasms. (C) Representative images illustrating fluorescence recovery of Alyref-GFP after photobleaching in the nuclear speckle (red circle) of live HeLa cells. (D) The plots of time-dependent normalized fluorescence recovery of Alyref-GFP in the nuclear speckle and nucleoplasm. RFU, relative fluorescent units; FRAP, fluorescence recovery after photobleaching; Scale bars, 5 µm.

To characterize the dynamic behavior of mRNA export proteins in different compartments of the nucleus, we used the fluorescence recovery after photobleaching (FRAP) technique. We measured the half-life of recovery and mobility of mRNA export proteins tagged with GFP within the nuclear speckles and nucleoplasm of live HeLa cells. Chtop-GFP present in the nuclear speckles had a half life for recovery from photobleaching of 141.46 seconds (Table 1) and reached a plateau of fluorescence intensity post-bleaching after 800 seconds (Figure 8A,B). In contrast, Chtop-GFP in the nucleoplasm showed a post-bleach half life for recovery of 48.47 seconds and reached a plateau of fluorescence intensity within 300 seconds (Figure 8B). These data indicate that Chtop-GFP is significantly less mobile in nuclear speckles than in the nucleoplasm. Earlier FRAP studies on whole nuclei for Chtop-GFP also showed that it took in excess of 800 seconds to reach a post-bleach plateau of fluorescence recovery [20] which fits with the observation that a large proportion of Chtop-GFP resides in the nuclear speckles. A large fraction of Chtop-GFP was found in the immobile fractions in both the nucleoplasm (39.23%) and nuclear speckles (43.95%) (Table 1), which for nucleoplasmic Chtop is consistent with the observation that it stably associates with chromatin [20]. We also analysed the mobility of two Chtop-GFP fragments comprising residues 1–87 and 93–213 in the nucleoplasm, and found that both these fragments of Chtop had substantially shorter half lives for recovery and lower immobile fractions compared with full length Chtop (Table 1), suggesting that Chtop requires both N and C-terminal regions for interaction with nuclear structures which reduce its mobility. For comparison we also monitored the mobility of two other export factors, Alyref and Nxf1 (Figure 8C, D and Table 1). Alyref-GFP and Nxf1-GFP have very short half lives for recovery post-bleaching compared with Chtop-GFP and much smaller immobile fractions, indicating that these two export factors have far greater mobility within the nucleus than Chtop. Interestingly, Alyref showed reduced mobility in the nuclear speckles compared with the nucleoplasm as did Chtop (Table 1). The increased mobility of Chtop and Alyref within the nucleoplasm compared with the nuclear speckles is consistent with the NFRET and FLIM-FRET data which show that a major site for assembly of TREX with Nxf1 occurs around nuclear speckles. Therefore a proportion of the nucleoplasmic Chtop and Alyref may be molecules already assembled with TREX and Nxf1, in the process of export to the cytoplasm.

**Table 1:** FRAP measurement of export factors in living cells.

Plasmid^1^	Location	Half-life of recovery (s^2^)	Immobile fraction	n^3^
Chtop	Speckle	141.46	43.95±4.21%	7
	Nucleoplasm	48.47	39.23±1.38%	7
Chtop 1–87	Nucleoplasm	3.92	7.73±2.29%	9
Chtop 93–213	Nucleoplasm	2.44	13.56±3.69%	8
Alyref	Speckle	2.42	17.18±1.29%	8
	nucleoplasm	0.65	13.52±3.30%	6
Nxf1	nucleoplasm	1.43	14.40±2.67%	7

1 Constructs with GFP fusion protein,

2 s time of half-life of recovery,

3 n number of cells from which the FRAP is measured.

## Discussion

We have spatially mapped the site where TREX subunits associate with each other and the mRNA export receptor Nxf1. We found that the major sites for interaction between two TREX subunits and Nxf1 do not lie within nuclear speckles. This is despite observing significant a concentration of TREX subunits within nuclear speckles. Instead, the major interaction sites are often found in close proximity to the nuclear speckles and these sites of interaction are sensitive to transcriptional inhibition. These data are consistent with an earlier study which used bifluorescence complementation to look at the interaction between Nxf1 and the exon-junction complex component Y14 [21]. In these earlier studies, Nxf1 and Y14 interactions were prominently observed in perispeckle regions, surrounding nuclear speckles in some cells, though weaker interactions were also observed within speckles. We also provide further evidence confirming that methylation of Chtop provides an important control step during assembly and maturation of the mRNA export complex. We have shown that unmethylated Chtop is able to bind Alyref *in vivo* (Figure 7E). This interaction probably occurs early during TREX assembly since only methylated Chtop subsequently binds Nxf1 within assembled TREX (Figure 2C and [5]).

TREX assembly is coupled with transcription, splicing and polyadenylation, moreover Nxf1 recruitment to mRNA requires an assembled TREX complex [2]. Thus Nxf1 recruitment to TREX might be expected to occur at sites where these processes take place. Nuclear speckles are devoid of DNA and transcription does not take place within the majority of them [22], though a number of proteins involved in transcription are found associated with nuclear speckles [8]. Active transcription sites are found at many sites within the nucleus but are enriched in regions surrounding nuclear speckles known as perispeckles [23]. Perispeckles correspond to a doughnut like ring structure found immediately surrounding nuclear speckles and these are the assembly site for the exon junction complex (EJC). This observation fits nicely with the earlier observation that splicing factors feed out from nuclear speckles to adjacent sites of transcription [13] since the EJC assembles on spliced RNA as does TREX. Whilst we have not observed the tight clustering of TREX:Nxf1 assembly sites around nuclear speckles in the same way that EJC components assemble in the perispeckle region, we clearly see extensive interactions in the perispeckle region. Thus the perispeckle region appears to be a major site for both assembly of TREX and subsequent recruitment of Nxf1.

When we inhibit transcription there is a significant reduction in Nxf1:TREX interactions observed within the nucleus, which suggests that TREX assembly with Nxf1 might occur cotranscriptionally. Consitently, the TREX subunits Hpr1 and Alyref are recruited cotranscriptionally [24] [25] [26]. The sites of Nxf1:TREX interaction which we observe which are not tightly associated with nuclear speckles may correspond to additional sites of transcription within the nucleus or TREX:Nxf1:mRNA complexes in transit to the cytoplasm. The other striking observation following the inhibition of transcription is the persistence of TREX subunit:Nxf1 interactions at the nuclear periphery. This interaction may represent complexes which have become trapped at the nuclear pore following the transcription block. In yeast, proofreading of mRNPs at the nuclear pore involves the Mlp proteins which can feedback and alter gene transcription in response to defects in the mRNP [27] and such a proofreading mechanisms exists in human cells involving the mammalian orthologue of Mlp1, which is Tpr [28]. Such a proofreading mechanism involving might also stall some preassembled mRNPs at the nuclear pore when transcription is inhibited. Nxf1 itself appears to stably associate with the nuclear pore since detergent extraction of cells prior to fixation readily reveals a distinctive nuclear rim association for Nxf1, though this association is dependent on the TREX complex [2]. Thus it seems likely that the interaction which persists at the nuclear pore involves assembled TREX association with Nxf1.

TREX deposition on mRNA is coupled with splicing [29] and the majority of splicing occurs co-transcriptionally. The majority of active spliceosomes localize to the periphery of nuclear speckles [16] and we see extensive TREX subunit and Nxf1:TREX interactions at the periphery of nuclear speckles which provides further support for the idea that TREX assembly is coupled with splicing. However, approximately 15–20% of active spliceosomes are found within nuclear speckles and these are engaged in post-transcriptional splicing [16]. Post-transcriptionally spliced mRNA within nuclear speckles require mRNA export factors including Alyref and Uap56 to exit the nuclear speckle domain and for subsequent export to the cytoplasm [16] [17]. Using FLIM-FRET, we are able to see interactions between TREX subunits and TREX:Nxf1 within speckles, indicating that active mRNA export complexes do form within this domain, but the number of molecules engaged in these is much lower than at the periphery of speckles. This probably reflects the low percentage of transcripts which undergo post-transcriptional splicing.

In conclusion we have provided the first spatial map of mRNA export complex assembly by investigating where TREX assembly takes place and where Nxf1 is recruited to TREX in vivo. The major sites of mRNA export complex assembly coincide with sites involved in transcription, splicing and exon junction complex formation revealing how intimately coupled these processes are likely to be.
